# Unmet contraceptive needs among female sex workers (FSWs) in semi urban Blantyre, Malawi

**DOI:** 10.1186/s12978-020-01064-w

**Published:** 2021-01-19

**Authors:** Donatien Twizelimana, Adamson S. Muula

**Affiliations:** 1 Ekwendeni Mission Hospital, P.O. Box: 19, Ekwendeni, Mzimba, Malawi; 2 Department of Public Health, College of Medicine, School of Public Health and Family Medicine, University of Malawi, Private Bag 360, Chichiri, Blantyre, Malawi; 3 The Africa Centre of Excellence in Public Health and Herbal Medicine (ACEPHEM) College of Medicine, University of Malawi, Private Bag 360, Chichiri, Blantyre, Malawi

**Keywords:** Unmet needs, Female sex workers, Contraception, Semi urban blantyre, Malawi

## Abstract

**Background:**

Research has paid limited attention to understanding factors that are associated with unmet contraceptive needs among female sex workers. In order to fill this knowledge gap, we estimated the prevalence of unmet contraceptive needs and examined associated factors among FSWs in semi urban Blantyre, Malawi.

**Methods:**

We used systematic sampling to recruit 290 female sex workers in semi urban Blantyre between February and March 2019. In this cross sectional study, we used questionnaire interviews to collect quantitative data. We calculated the mean and standard deviation for continuous variables and proportions for categorical variables to describe the data. Logistic regression analysis was used to investigate the association between unmet needs (the outcome variable) and explanatory variables such as: having a steady partner, fear of contraceptives’ side effects and having a history of sexually transmitted infections.

**Results:**

Out of the 290 study participants 102 (35.2%) reported unmet contraceptive needs. The following factors were significantly associated with unmet contraceptive needs in multivariate analysis: female sex workers’ history of physical and sexual violence by clients [OR 3.38, 95% CI (1.10, 10.43)], p < 0.03, participants with a steady partner [OR 3.28, 95% CI (1.89, 5.68)], p < 0.001, and participants who feared side effects of contraceptives [OR 2.99, 95% CI (1.73, 5.20)], p < 0.001.

**Conclusion:**

Reproductive Health services should address barriers to contraceptives use for instance: violence by female sex workers’ clients, fear and misinformation on contraceptives. There is need to improve awareness of contraceptives. Specific health promotion interventions on female sex workers engaged in a steady partnership are recommended. It is important to enhance the knowledge, attitudes, and counseling skills of health care providers in order to address unmet contraceptive needs among female sex workers in semi-urban Blantyre.

**Plain English summary:**

Unmet contraceptive needs are defined as lack of contraceptives use in heterosexually active women of childbearing age who do not wish to become pregnant. Unmet contraceptive needs are the main cause of short inter-pregnancy intervals, early childbearing, physical abuse, unintended pregnancy, poor maternal and child health outcomes. Several studies have documented low contraceptives use among female sex workers (FSWs), but research has paid limited attention to understanding factors associated with unmet contraceptive needs among this population in semi urban Blantyre Malawi.

In order to fill this knowledge gap, we estimated the prevalence of unmet contraceptive needs and examined factors that were associated with unmet contraceptive needs among FSWs in semi urban Blantyre, Malawi. We recruited 290 FSWs and collected quantitative data. These data were analyzed to obtain descriptive statistics. Logistic regression analysis was used to investigate the association between unmet contraceptive needs (the outcome variable) and explanatory variables such as: FSWs with history of physical and sexual violence by clients, having a steady partner, fear of contraceptives’ side effects and having a history of sexually transmitted infections.

Out of the 290 FSWs, 35% reported unmet contraceptive needs. The following factors were significantly associated with unmet contraceptive needs in multivariate analysis: FSWs’ history of physical and sexual violence by clients, participants with a steady partner and participants who feared contraceptive side effects.

Sexual and Reproductive Health services should address barriers to contraceptives use, female sex workers exposure to violence, having a steady partners and concerns about side effects. There is also a need to improve the knowledge, attitudes, and counseling skills of health providers in order to address unmet contraceptive needs among FSWs.

## Background

Research has paid limited attention to understanding the prevalence of, and factors associated with unmet contraceptive needs among female sex workers (FSWs) [1–3]. There is often low contraceptives use among female sex workers as documented by several studies [4–7]. Many FSWs have intention to prevent pregnancy or to delay it [1, 2] but are limited by individual, political, societal, cultural, and health system challenges which converge to restrict access to contraceptives [2, 3, 8, 9]. Those barriers contribute to unmet contraceptive needs which are referred to as lack of contraceptives use in heterosexually active women of childbearing age who do not wish to become pregnant [3, 10]. Many studies found that FSWs face significant challenges in ensuring proper use of contraceptives such as hormonal contraceptives and condoms [1, 10]. Other than low contraceptives use, little is known about FSWs contraceptive preferences [1, 10]. Yet, unmet contraceptive needs are the main cause of short inter-pregnancy intervals, early childbearing, physical abuse, poor maternal and child health outcomes [11–13]. High female illiteracy, poverty and gender inequality are also associated with unsatisfied demand for contraceptives [14, 15].

Female sex workers operate in a very complex environment different from other women of reproductive age. They experience consistent and transient multiple partnerships [16]. Inconsistent condom use, high HIV prevalence and unintended pregnancies have also been reported among this hard to reach key population [16]. The challenges to access contraception services contribute to unintended pregnancies which in many cases are intentionally terminated [17]. Evidence in the literature suggests that there is increased maternal mortality resulting from unsafe abortions which are prevalent among FSWs in many countries [18]. Lack of access to contraceptives may also contribute to continued dependence on sex work in order to support children with daily needs, and HIV transmission to children if prevention of mother-to-child transmission services are not utilized [19].

Unmet contraceptive needs for family planning are public health concerns and key global health priority [15, 20]. Lack of contraceptives adversely affect many women of reproductive age [15, 20, 21]. In Malawi the total demand for contraceptives use among currently married women aged between 15–49 years old has increased over time, rising from 50% in 1992 to 61% in 2000, 63% in 2004, 72% in 2010, and 78% in 2015–16 [22]. As a result, unmet contraceptive needs among currently married women have been declining over the years, from 37% in 1992 to 19% in 2015- 16 [22]. Many studies have assessed contraceptives use among women of reproductive age in Malawi and sub-Saharan Africa. However, research has paid limited attention to understanding factors associated with unmet contraceptive needs among FSWs in semi urban Blantyre. Findings are expected to guide policy makers responsible in designing programs to improve FSWs’ health.

## Methods

The study was conducted between February and March 2019. In this cross sectional study, we collected quantitative methods on unmet contraceptive needs and preferences among FSWs in four semi urban townships of Blantyre (Chirimba, Lunzu, Kachere, Mbayani) of southern Malawi.

The following inclusion criteria were used: self-reported female sex workers aged between 18 and 49 years and consented for the study. We used the following exclusion criteria: female sex workers participating in another study or coming from outside the study locations, and FSWs who were sick.

### Sample size and calculation

The sample size was calculated using single population proportion formula. We used 25% as the prevalence of FSWs with unmet contraceptive needs [23], with a margin error of 5%, and at 95 level of confidence. Thus the required sample size was 288.

### Data collection process

At each of the four sites, data collection took place at places mutually agreed by the study team and research participants. About 75 FSWs from each site were systematically selected to participate in the study (we recruited FSWs who arrived at the study site on even days of the month). A specific period was identified within which the questionnaires were completed by the research assistants through the interview process.

Female research assistants trained in data collection and research went to each site at a pre-arranged time. They explained the purpose of the study and emphasized the fact that FSWs who do not wish to participate may either leave, or remain as study participants, but they will not be adversely affected by their voluntary decisions if they decided not to participate. Following the explanation, study participants were given an opportunity for them to ask questions and get clarifications. Data were collected on: socio-economic/demographic background of the informant, contraceptive preferences, access to contraceptives and preference of contraceptives providers.

One of the strengths of this study was the sample size of almost 300 FSWs which was used to explore several variables independently associated with unmet contraceptive needs. Secondly the study used validated questions on contraceptive use that are also employed by Malawi Demographic and Health Survey (MDHS) which will facilitate comparison to other literature. Each of the FSWs was asked questions by the research assistants and the survey questionnaires were completed by the data collectors. Cash reimbursement of Malawi Kwacha (MK) 1,500 (approximately 2 US$ at the time of data collection) was paid to all study participants as compensation for their time in the study.

### Data analysis

Data analysis was conducted in Stata 14.1 (Stata Corporation, College Station, TX, USA). Descriptive statistics were calculated from general socio-economic and demographic characteristics of the study participants. Logistic regression analysis was used to investigate the relationship between unmet contraceptive needs (the outcome) and hypothesized explanatory variables. Characteristics of participants were analyzed using frequency summaries and are presented as percentages. Age was summarized using median and interquartile range. Table 1 indicates the variables which were summarized. Note that steady partner attitude on contraceptives has two categories: FSWs with and without steady partners. The numbers and percentage of each category including the category of FSWs without steady partners were summarized in the baseline characteristics (Table 1). However, when performing univariate analysis to assess associations between the outcome and the independent variables, the category of FSWs without steady partners was excluded as the output from such a category did not have a meaningful interpretation with respect to associations with baseline variables.

**Table 1:** Characteristics of female sex workers in semi – urban Blantyre 2019 (N = 290)

Characteristics	N = 290
	Frequency (%)
Age in years, median (IQR)	25 (19.30)^a^
Education	
Primary	195 (67.2)
Secondary	84 (29.0)
Tertiary	10 (3.5)
None	1 (0.3)
Place of birth	
Rural	167 (57.6)
Urban	123 (42.4)
Other characteristics	
Consistent condom use	192 (66.2)
Additional income	66 (22.8)
Steady partner attitude for contraceptives (Positive)	35 (34.0)
Sexually transmitted infections	49 (16.9)
Physical and sexual violence by clients	61 (21.0)
Regular alcohol Intake	224 (77.2)
Steady partner	95 (32.8)
Poor knowledge of contraceptives	167 (57.6)
More than three living children	109 (37.6)
Child younger than one year	73 (25.2)
FSWs desired to have few children	195 (67.2)
Fear of side effects of contraceptives	150 (51.7)
Unmet needs for family planning	102 (35.2)

^a^ Median and IQR have been reported

### Operational definitions

In this study consistent condom use was defined as using condoms correctly for every act of penile-vaginal sex [24]. Regular alcohol intake was defined as having a minimum of one drink per day. This refers to the amount consumed on any single day and is not intended as an average over several days [25]. Steady partner was defined as spouse or cohabiting partner or someone with a romantic relationship with for a long period of time [24].

### Ethical consideration

The study was approved by the University of Malawi’s COMREC (College of Medicine Research and Ethics Committee). Certificate number P.07/18/2444, dated 08-Sept-2018. Blantyre District Office approved the study. We got clearance from the group village headmen before the study started. All study staff were carefully trained in human subjects’ protection, especially the importance of protecting privacy and confidentiality and obtaining informed consent from each study participant using the approved consent forms. Participants were informed of their right to withdraw from the study and not to answer any questions they felt uncomfortable with. All the information which was provided by the participants was treated with confidentiality.

## Results

### Female sex workers’ characteristics

We recruited a total of 290 study participants of which 66.2% reported consistent condom use. Ninety-five participants (32.8%) had steady partners. Positive steady partners’ attitude for contraceptives was observed in 34% of the participants who had steady partners. About 25.2% had a child younger than one year. Physical and sexual violence by clients were reported in 21% of the participants. There was a very high proportion of regular alcohol intake that accounted for 77.2% One hundred and ninety-five (67.2%) FSWs interviewed had attended only primary school as their highest level of academic achievement **(**Table 1).

We also found that male condom is the most preferred among FSWs in semi-urban Blantyre, while the IUCD is the least used (Table 2).

**Table 2:** Distribution of contraceptive prevalence among FSWs in semi urban Blantyre

Methods used	(N = 290) Frequency (%)
Oral Pills	20 (7.9)
IUCD	2 (0.7)
Injectable	30 (10.3)
Implant/Norplant	16 (5.5)
Female Condoms	5 (1.7)
Male condoms	81 (27.9)
Duo method^a^	126 (43.4)
Sterilization (Tubal ligation)	10 (3.4)
Natural method	0 (0)

^a^FSWs using either oral pills, IUCD, injectable contraceptives, and implants but are also using condoms for HIV, and STI prevention

We assessed associations between unmet contraceptive needs and independent variables firstly by using the univariate logistic regression model. All variables that were significant in the univariate analysis were included in the multivariable regression model. Both the unadjusted and adjusted odds ratios, the confidence intervals and p values from the logistic regression model are reported. Statistical significance was declared at 5% significance level. All analyses were performed using Stata 14.

In the univariate logistic regression model (Table 3), we found four variables which were significantly associated with unmet contraceptive needs. Participants with history of sexually transmitted infections were 3 times more likely to have unmet contraceptive needs compared to those without OR 3.34, 95% CI (1.77, 6.28) p < 0.001. Participants who had a history of physical and sexual violence by clients were about 4 times more likely to have unmet needs for family planning compared to those without OR 3.56, 95% CI (1.98, 6.38), p < 0.001. Participants with steady partners were about 4 times more likely to have unmet contraceptive needs compared to those without OR 3.76, 95% CI (2.24, 6.31), p < 0.001. Participants who reported fearing side effects of contraceptives were about 3 times more likely to have unmet contraceptive needs compared to those without the same fear OR 2.95, 95% CI (1.78, 4.91), p < 0.001) (Table 3).

**Table 3:** Univariate logistic regression: associations between unmet contraceptive needs and independent variables

Unmet contraceptive needs	Odds ratio	[95% CI]	*P*-value
Education			
Secondary	0.68	0.40, 1.18	0.17
Tertiary	0.40	0.08, 1.93	0.26
Primary	1.00	Ref	
Other characteristics			
Consistent condom use (Yes)	0.74	0.45, 1.22	0.24
Additional income (Yes)	0.82	0.46, 1.48	0.52
Steady partner attitude for contraceptives (YES)	1.41	0.62, 3.23	0.41
Sexually Transmitted Infections (YES)	3.34	1.77, 6.28	< 0.001
Physical and sexual violence by clients (YES)	3.56	1.98, 6.38	< 0.001
Regular alcohol Intake (YES)	1.21	0.68, 2.18	0.52
Steady partner (YES)	3.76	2.24, 6.31	< 0.001
Poor knowledge of contraceptives (YES)	0.95	0.59, 1.55	0.85
More than three living children (YES)	0.66	0.40, 1.10	0.11
Child younger than one year (YES)	1.40	0.81, 2.42	0.22
FSWs who desired to have few children (YES)	0.78	0.47, 1.30	0.35
Fear of side effects of contraceptives (YES)	2.95	1.78, 4.91	< 0.001

### Multivariable logistic regression

The four variables that were significantly associated with unmet contraceptive needs in the univariate analyses were: sexually transmitted infections status, history of physical and sexual violence by partner’clients status, steady partner and fear of side effects of contraceptives status were included in the multivariable logistic regression analysis. As shown in the Table 4 below, all of them were significantly associated with unmet contraceptive needs, except sexually transmitted infections. In the multivariable analysis participants with physical and sexual violence by clients were about 3 times more likely to have unmet contraceptive needs compared to those without OR 3.38, 95% CI (1.16, 10.43), p < 0.03. Participants with a steady partner were about 3 times more likely to have unmet contraceptive needs compared to those without OR 3.28, 95% CI (1.89, 5.68), p < 0.001. Participants who reported being afraid of side effects of contraceptives were about 3 times more likely to have unmet contraceptive needs compared to those without OR 2.99, 95% CI (1.73, 5.20), p < 0.001.

**Table 4:** Multivariable logistic regression: associations between unmet contraceptive needs and independent variables

Unmet contraceptive needs	Odds ratio	95% CI	P-value
Sexually transmitted infections (Yes)	1.05	0.31, 3.55	0.94
Physical and sexual violence by clients (Yes)	3.38	1.16, 10.43	0.03
Steady partner (Yes)	3.28	1.89, 5.68	< 0.001
Fear of side effects of contraceptives (Yes)	2.99	1.73, 5.20	< 0.001

## Discussion

We investigated the prevalence of unmet contraceptive needs and its association with selected explanatory variables among FSWs in semi urban Blantyre, Malawi. Despite the desire to have few children, there is a high prevalence of unmet contraceptive needs among FSWs in the study setting. Out of 290 FSWs, 102 (35.2%) reported having unmet contraceptive needs. This is higher than the usually reported prevalence of unmet contraceptive needs among women of reproductive age in Malawi, which is estimated to be 19% [22]. The difference between our estimates and the Malawi Demographic and Heath Survey (MDHS) estimates may be due to different populations studied with our study having recruited FSWs while the MDHS recruited women in the general population. High prevalence of unmeet contraceptive needs can also be explained by FSWs fear of their side effects which is mainly related to poor knowledge of different types of contraceptives [2, 3]. Our findings suggest that FSWs who reported being afraid of side effects of contraceptives were about 3 times more likely to have unmet needs for contraceptives compared to those without OR 2.99, 95% CI (1.73, 5.20), p < 0.001. Our study findings suggest that more than a half (57%) of the population studied has poor knowledge of contraceptives. Our findings are similar to a study done in Kenya. [26]. In the Kenyan study the authors reported that side effects such as spotting, dizziness, nausea interfered with FSWs business of sex trade. This suggested that FSWs may avoid using contraceptives due to the fear of losing clients and consequently, income [26, 27].

Studies also suggest that the use of non-barrier contraceptives methods among FSWs is estimated to be lower than 40% across many countries in sub-Saharan Africa [17, 28, 29]. Oral pills and injectable contraceptives were reported as the most commonly used by FSWs. Although these methods are effective when used consistently, reports suggest that they have high discontinuation rates due to side effects, and daily adherence to oral pills is most of times difficult [30–33]. Our study findings suggest that LARCs (Long-acting reversible contraceptives) IUCDs (intra-uterine contraceptive devices), implants/ norplants were less preferred by our study participants. This is consistent with studies done elsewhere [30–34]. LARCs are highly recommended globally as effective contraceptives, have less side effects as compared to other methods but are rarely used by FSWs in many sub-Saharan Africa due to non-availability and utilization problems [34].

With low contraceptives use among FSWs there is high risk of unintended pregnancy among FSWs which in most cases result in termination [35, 36]. In countries where abortion laws are not explicit or are restrictive, FSWs opt for unsafe abortions which have unfavorable results [35, 36]. Studies suggest that in sub-Saharan African unsafe abortions contribute to 10% of maternal deaths [37–39]. Fear and misinformation of contraceptives can be addressed by initiating and increasing awareness [26, 27, 40]. Improving providers counseling skills was also reported to be effective in improving FSWs contraceptive uptake. Targeted outreach services may help overcome some of these considerable challenges among FSWs in semi urban Blantyre. The future direction to address maternal death should target all women of reproductive age including FSWs. Public health interventions such as improving the uptake of contraceptives may avert many cases of maternal death in this community and other women of reproductive age.

Contrary to many other studies we did not find an association between access to family planning and unmet contraceptive needs. This can be explained by availability of free contraceptives services in public and some private institutions in Malawi. Overall, the contraceptive prevalence rate (CPR) is 59% for currently married women age 15–49 years. Among sexually active, unmarried women age15-49, 44% use a contraceptive method and 43% use a modern method [22]. Another explanation is that male condoms are mostly used for contraception and/or as dual method and are easily found free of charge from government, private institutions and in many shops at affordable prices. Out of 290 FSWs, 81 (27.9%) reported to use condom primarily for contraception and 126 FSWs (43.4%) reported to use condom as dual method (contraceptive and prevention of sexually transmitted infections).

Despite the wide availability of male condoms, only 192 FSWs (66.2%) reported to use them consistently. This reflects that most likely there’s inconsistent condom use among the remaining study participants and their steady partners. In this study we found that 95 FSWs (32.7%) have steady partners. Participants with steady partners were about 3 times more likely to have unmet contraceptive needs compared to those without OR 3.28, 95% CI (1.89, 5.68), *p* < 0.001. Condomless sex put FSWs and their steady partners at high risk of HIV and STI as well as pregnancies [24, 26, 41, 42]. In qualitative studies done elsewhere FSWs reported not using condoms with their boyfriends and/or steady partners reflect intimacy and trust which are perceived to contribute to stronger relationships [24, 41–46]. Inconsistent condom use, condom failure and considerations for income were reported as prevalent issues among FSWs in many study settings [47–49]. These findings highlight a critical need to provide education to improve condom availability, access and uptake among FSWs. There is also a need to improve the knowledge, attitudes and counseling skills of health providers. For effective contraception, the dual method referred to the use of condom with an effective non-barrier method for both HIV/STI and pregnancy prevention is recommended [50].

We found that female sex workers with history of physical and sexual violence by clients were about 3 times more likely to have unmet contraceptive needs compared to those without, OR 3.38, 95% CI (1.16, 10.43), *p* < 0.03. This is consistent with studies done elsewhere [51–53]. Sexual and gender based violence have adverse effects on reproductive health of female sex workers. This study found that 61% of the study participants reported to have experienced physical, gender and sexual violence. Pertinent interventions with inputs from the community, health, police and legal sector are required to address this issue.

## Limitations of the study

Since sex work is a sensitive issue, there is a possibility of social desirability bias which in turn underestimates the magnitude of studied matter. Care was taken to assure the study participants of confidentiality of their information and privacy. These actions potentially minimized reporting biases. Secondly, this was a cross-sectional study, it does not provide evidence of a temporal relationship between exposures and unmet contraceptive needs. The possibility that the associations observed between unmet contraceptive needs and the selected explanatory variables may be context-dependent and not necessarily applicable to other countries [8]. Further research should be conducted to assess the reproducibility of these relationships. It is also possible that the additional explanatory variables not found in this study may also be relevant for unmet contraceptive needs among FSWs in the same study location. There is also a possibility of recall bias and misreporting of personal experiences [8].

## Conclusion

We found that about 35.2% female sex workers in semi-urban Blantyre, Malawi had unmet contraceptive needs. More than a half (57%) had poor knowledge of contraceptives. Our findings further indicate that physical and sexual violence by partner, having steady partners, fear of contraceptives side effects are independently associated with unmet contraceptive needs among the study participants. There is a critical need to improve awareness of contraceptives among FSWs in semi urban Blantyre. Sexual and reproductive health services should also address other barriers to contraceptives use such as partner violence, and misinformation on contraceptives. There is also a need to improve the knowledge, attitudes and counseling skills of health providers in order to address unmet contraceptive needs among FSWS.

## Data Availability

The datasets used and/or analyzed during the current study are available from the corresponding author on reasonable request.

## Abbreviations

ACEPHEM: Africa Center of Excellence in Public Health and Herbal Medicine
ART: Antiretroviral therapy
AOR: Adjusted odds ratio
CI: Confidence interval
CPR: Contraceptive prevalence rate
COMREC: College of Medicine Research and Ethics Committee
FSWs: Female sex workers
IUCD: Intrauterine contraceptive device
IQR: Interquartile range
LARC: Long acting reversible contraceptives
NGO: Non-governmental organization
MDHS: Malawi Demographic and Health Survey
OR: Odds ratio
SRH: Sexual and reproductive health
STI: Sexual transmitted infection

## References

[1] Kenya National Bureau of Statistics (KNBS) and ICF Macro (2010). Kenya Demographic and Health Survey 2008–09.

[2] Bankole A, Hussain R, Sedgh G, Rossier C, Kaboré I, Guiella G (2014). Unintended pregnancy and induced abortion in Burkina Faso: Causes and consequences.

[3] Nortman DL (1982). Measuring the unmet need for contraception to space and limit births. Int Fam Plan Perspect.

[4] Wayal S, Cowan F, Warner P, Copas A, Mabey D, Shahmanesh M (2011). Contraceptive practices, sexual and reproductive health needs of HIV-positive and negative female sex workers in Goa India. Sex Transm Infect.

[5] Todd CS, Alibayeva G, Sanchez JL, Bautista CT, Carr JK, Earhart KC (2006). Utilization of contraception and abortion and its relationship to HIV infection among female sex workers in Tashkent Uzbekistan. Contraception.

[6] Todd CS, Nasir A, Raza Stanekzai M, Scott PT, Strathdee SA, Botros BA, Tjaden J (2010). Contraceptive utilization and pregnancy termination among female sex workers in Afghanistan. J Womens Health (Larchmt).

[7] Khan MR, Turner AN, Pettifor A, Van Damme K, Rabenja NL, Ravelomanana N, Sweezey T, Williams D, Jamieson D, Mad STI Prevention Group, Behets F. Unmet need for contraception among sex workers in Madagascar. *Contraception.* 2009;79(3):221–7. 10.1016/j.contraception.2008.09.011 PMC5817995 19185677

[8] Blanc AK, Way AA (1998). Sexual behavior and contraceptive knowledge and use among adolescents in developing countries. Stud Fam Plan.

[9] Casterline JB, Sinding SW (2000). Unmet need for family planning in developing countries and implications for population policy. Popul Dev Rev.

[10] Westoff CF, Pebley AR (1981). Alternative measures of unmet need for family planning in developing countries. Int Fam Plan Perspect.

[11] Genet E, Abeje G, Ejigu T. Determinants of unmet need for family planning among currently married women in Dangila town administration, Awi Zone, Amhara regional state; a cross sectional study. Reproductive Health. 2015;12(1):42. 10.1186/s12978-015-0038-3 PMC4436105 25962743

[12] Sedgh G, Singh S, Hussain R (2014). Intended and unintended pregnancies worldwide in 2012 and recent trends. Stud Fam Plann.

[13] EDHS. Key indicators report for Ethiopia demographic and health survey: the DHS program ICF Rockville, Maryland, USA. . Report. Ethiopia: 2016

[14] Kabagenyi A, Jennings L, Reid A, Nalwadda G, Ntozi J, Atuyambe L. Barriers to male involvement in contraceptive uptake and reproductive health services. A Qualitative Study of Men and Women’s Perceptions in Two Rural Districts in Uganda. Reprod Health. 2014;11:21. 10.1186/1742-4755-11-21 PMC3946591 24597502

[15] Mosha I, Ruben R, Kakoko D (2013). Family planning decisions, perceptions and gender dynamics among couples in Mwanza, Tanzania. A Qualitative Study. BMC Pub Health.

[16] Muula A, Twizelimana D (2015). HIV and AIDS risk perception among sex workers in semi-urban Blantyre, Malawi. Tanzania J Health Res.

[17] Schwartz S, Papworth E, Thiam-Niangoin M, Abo K, Drame F, Diouf D (2009). An urgent need for integration of family planning services into HIV care: the high burden of unplanned pregnancy, termination of pregnancy, and limited contraception use among female sex workers in Coˆte d'Ivoire. J Acquir Immune DeficSyndr.

[18] Yam EA, Mnisi Z, Mabuza X, Kennedy C, Tsui A, Baral S (2013). Use of dual protection among female sex Workers in Swaziland. IntPerspect Sex Reprod Health.

[19] Chanda MM, Ortblad KF, Mwale M, Chongo S, Kanchele C, Kamungoma N, Barresi LG, Harling G, Bärnighausen T, Oldenburg CE (2017). Contraceptive use and unplanned pregnancy among female sex workers in Zambia. Contraception.

[20] Cleland J, Bernstein S, Ezeh A, Faundes A, Glasier A, Innis J (2006). Family planning: the unfinished agenda. Lancet.

[21] Upadhyay UD, Gipson JD, Withers M, Lewis S, Ciaraldi EJ, Fraser A, Huchko MJ, Prata N (2014). Women's empowerment and fertility: a review of the literature. SocSci Med.

[22] National Statistics Office (NSO) [Malawi] and ICF International. Malawi Demographic and Health Survey 2015–2016: Key Indicators Report. Zomba, Malawi and Rockville, Maryland: NSO and ICF International; 2016.

[23] Long JE, Waruguru G, Yuhas K, Wilson KS, Masese LN, Wanje G, Kinuthia J, Jaoko W, Mandaliya KN, McClelland RS (2019). Prevalence and predictors of unmet contraceptive need in HIV-positive female sex workers in Mombasa, Kenya. PLoS ONE.

[24] Weldegebreal R, Melaku YA, Alemayehu M, Gebrehiwot TG (2015). Unintended pregnancy among female sex workers in Mekelle city, northern Ethiopia: a cross-sectional study. BMC Public Health.

[25] Chan AM, von Mühlen D, Kritz-Silverstein D, Barrett-Connor E (2009). Regular alcohol consumption is associated with increasing quality of life and mood in older men and women: the Rancho Bernardo Study. Maturitas.

[26] Sutherland EG, Alaii J, Tsui S, Luchters S, Okal J, Kingola N, Temmerman M, Jannowitz B (2011). Contraceptive needs of female sex workers in Kenya—a cross-sectional study. Eur J ContraceptReprod Health Care.

[27] Boudreau CL (2015). Assessing the contraceptive needs of female sex workers in Kigali, Rwanda. Ann Global Health.

[28] Lafort Y, Greener R, Roy A, Greener L, Ombidi W, Lessitala F, Skordis-Worrall J, Beksinska M, Gichangi P, Reza-Paul S, Smit JA, Chersich M, Delva W (2017). Sexual and reproductive health services utilization by female sex workers is context-specific: results from a cross-sectional survey in India, Kenya, Mozambique and South Africa. Reprod Health.

[29] Surie D, Yuhas K, Wilson K, Masese LN, Shafi J, Kinuthia J, Jaoko W, McClevellan RS (2017). Association between non-barrier modern contraceptive use and condomless sex among HIV-positive female sex workers in Mombasa, Kenya: a prospective cohort analysis. PLoS ONE.

[30] Tolley E, Loza S, Kafafi L, Cummings S (2005). The impact of menstrual side effects on contraceptive discontinuation: findings from a longitudinal study in Cairo Egypt. IntFam Plan Perspect.

[31] Ampt FH, Lim MSC, Agius PA, Chersich MF, Manguro G, Gichuki CM, Stoove M, Tammerman M (2019). Use of long-acting reversible contraception in a cluster-random sample of female sex workers in Kenya. Int J GynaecolObstet.

[32] Morse J, Chipato T, Blanchard K (2013). Provision of long-acting reversible contraception in HIV-prevalent countries: results from nationally representative surveys in southern Africa. BJOG.

[33] AjongAB, Njotang PN, Kenfack B, Essi MJ, Yakum MN, Ibella FBS. Contraceptive method mix and preference: A focus on long acting reversible contraception in urban Cameroon. PLoS ONE. 2018;13(8):e0202967. 10.1371/journal.pone.0202967 PMC6107242 30138474

[34] Ampt FH, Willenberg L, Agius PA, Chersich M, Luchters S, Lim MSC (2018). Incidence of unintended pregnancy among female sexworkers in low-income and middle-income countries: a systematic review and meta-analysis. BMJ Open.

[35] Tebeu PM, Halle-Ekane G, DaItambi M, EnowMbu R, Mawamba Y, Fomulu JN (2015). Maternal mortality in Cameroon: a university teaching hospital report. Pan Afr Med J.

[36] Kamga DVT, Nana PN, Fouelifack FY, Fouedjio JH (2017). Role of abortion and ectopic pregnancies in maternal mortality rate at 3 university hospitals in Yaounde [inFrench]. Pan Afr Med J.

[37] Department of Reproductive Health and Research, World Health Organization (WHO). Unsafe abortion: global and regional estimates of the incidence of unsafe abortion and associated mortality in 2008. 6th ed. Geneva: WHO; 2008. https://www.who.int/reproductivehealth/publications/unsafe_abortion/9789241501118/en/. Accessed 13 Apr 2020.

[38] Say L, Chou D, Gemmill A, Tuncalp O, Moller AB, Daniels J, Gulmezoglu AM, Temmerman M, Alkma L (2014). Global causes of maternal death: a WHO systematic analysis. Lancet Glob Health.

[39] Grimes DA, Benson J, Singh S, Romero M, Ganatra B, Okonofua FE, Shah I (2006). Unsafe abortion: the preventable pandemic. Lancet.

[40] Wall KM, Vwalika B, Haddad L, Khu NH, Vwalika C, Kilembe W, Chomba E, Stephenson R, Kleinbaum D, Nizam A, Brill I, Tichacek A, Allen S (2013). Impact of long-term contraceptive promotion on incident pregnancy: a randomized controlled trial among HIV-positive couples in Lusaka Zambia. J Acquir Immune DeficSyndr.

[41] Thomsen SC, Ombidi W, Toroitich-Ruto C, Wong EL, Tucker HO, Homan R, Kingola N, Luchters S (2006). A prospective study assessing the effects of introducing the female condom in a sex worker population in Mombasa Kenya. Sex Transm Infect.

[42] Luchters, S, Richter, ML, Bosire, W, Nelson, G, Kingola, N, Zhang, X-D, Temmerman, M Chersich, MF. 'The contribution of emotional partners to sexual risk taking and violence among female sex workers in Mombasa, Kenya: a cohort study. PLoS ONE. 2013; 8(8):e68855. 10.1371/journal.pone.0068855 PMC3737234 23950879

[43] Warren CE, Mayhew SH, Hopkins J (2017). The current status of research on the integration of sexual and reproductive health and HIV services. Stud FamPlann.

[44] Ippoliti NB, Nanda G, Wilcher R (2017). Meeting the reproductive health needs of female key populations affected by HIV in low- and middle-income countries: a review of the evidence. Stud Fam Plann.

[45] Dhana A, Luchters S, Moore L, Lafort Y, Roy A, Scorgie F (2014). Systematic review of facility-based sexual and reproductive health services for female sex workers in Africa. Glob Health.

[46] Thyda L, Sineng S, Delvaux T, Srean C, Mary S, Vuochnea P (2015). Implementation and operational research: integration of family planning services in a peer-managed HIV care clinic serving most-at-risk populations in Phnom Penh Cambodia. J Acquir Immune DeficSyndr.

[47] Decker MR, Lyons C, Billong SC, Njindam IM, Grosso A, Nunez GT, Tumasang F, LeBreton M, Tamoufe U, Baral S (2016). Gender-based violence against female sex workers in Cameroon: prevalence and associations with sexual HIV risk and access to health services and justice. Sex Transm Infect.

[48] Lim S, Peitzmeier S, Cange C, Papworth E, LeBreton M, Tamoufe U, Baral S (2015). Violence against female sex workers in Cameroon: accounts of violence, harm reduction, and potential solutions. J Acquir Immune DeficSyndr.

[49] Abelson A, Lyons C, Decker M, Katende S, Njidam IM, Fouda G, Ndonko F, Levitt D, Tamoufe U, Billong S, Zoung-KanyiBissek AC, Balal S (2019). Lifetime experiences of gender based violence, depression and condom use among female sex workers in Cameroon. Int J Soc Psychiatry.

[50] World Health Organization(WHO), United Nations Population Fund, Joint United Nations Programme on HIV/AIDS, Global Network of Sex Work Projects, The World Bank. Implementing comprehensive HIV/STI programmes with sex workers: practical approachesvfromvcollaborativevintervention. Geneva: WHO; 2013. https://www.who.int/hiv/pub/sti/sex_worker_implementation/en/. Accessed 25 Apr 2020

[51] Wechsberg WM, Luseno WK, Lam WKK, Parry CDH, Morojele NK (2006). Substance use, sexual risk, and violence: HIV prevention intervention with sex workers in Pretoria. AIDS Behav.

[52] Okal JCM, Tsui S, Sutherland E, Temmerman M, Luchters S (2011). Sexual and physical violence against female sex workers in Kenya: a qualitative enquiry. AIDS Care.

[53] Kenya National Bureau of Statistics. Kenya Demographic and Health Survey 2014. 2015.

